# Interlinkage among cardio-metabolic disease markers in an urban poor setting in Nairobi, Kenya

**DOI:** 10.3402/gha.v9.30626

**Published:** 2016-02-09

**Authors:** Tilahun Nigatu Haregu, Samuel Oti, Nicholas Ngomi, Christopher Khayeka–wandabwa, Thaddaeus Egondi, Catherine Kyobutungi

**Affiliations:** 1 African Population and Health Research Center, Nairobi, Kenya; 2 Department of Global Health, Academic Medical Center, University of Amsterdam, Amsterdam, The Netherlands; 3 Amsterdam Institute for Global Health and Development, Amsterdam, The Netherlands

**Keywords:** interlinkage, cardio-metabolic markers, urban, sub-Saharan Africa

## Abstract

**Introduction:**

The main cardio-metabolic diseases – mostly cardiovascular diseases such as stroke and ischemic heart disease – share common clinical markers such as raised blood pressure and blood glucose. The pathways of development of many of these conditions are also interlinked. In this regard, a higher level of co-occurrence of the main cardio-metabolic disease markers is expected. Evidence about the patterns of occurrence of cardio-metabolic markers and their interlinkage in the sub-Saharan African setting is inadequate.

**Objective:**

The goal of the study was to describe the interlinkage among common cardio-metabolic disease markers in an African setting.

**Design:**

We used data collected in a cross-sectional study from 5,190 study participants as part of cardiovascular disease risk assessment in the urban slums of Nairobi, Kenya. Five commonly used clinical markers of cardio-metabolic conditions were considered in this analysis. These markers were waist circumference, blood pressure, random blood glucose, total blood cholesterol, and triglyceride levels. Patterns of these markers were described using means, standard deviations, and proportions. The associations between the markers were determined using odds ratios.

**Results:**

The weighted prevalence of central obesity, hypertension, hyperglycemia, hypercholesterolemia, and hypertriglyceridemia were 12.3%, 7.0%, 2.5%, 10.3%, and 17.3%, respectively. Women had a higher prevalence of central obesity and hypercholesterolemia as compared to men. Blood glucose was strongly associated with central obesity, blood pressure, and triglyceride levels, whereas the association between blood glucose and total blood cholesterol was not statistically significant.

**Conclusions:**

This study shows that most of the common cardio-metabolic markers are interlinked, suggesting a higher probability of comorbidity due to cardio-metabolic conditions and thus the need for integrated approaches.

## Paper in context

Cardio-metabolic diseases share risk factors and tend to co-occur, resulting in comorbidities. Though binary associations between markers of cardio-metabolic conditions have been assessed in the Western world, there is insufficient evidence about the interlinkage among them, particularly in sub-Saharan Africa. This study examined the interlinkage among five cardio-metabolic biomarkers and found a significant association. The evidence can inform integrated approaches to the prevention and control of common cardio-metabolic diseases.

## Introduction

The common non-communicable diseases (NCDs) – cardiovascular diseases (CVDs), cancers, and chronic respiratory diseases, which are on the increase in many low- and middle-income countries, share common clinical risk factors (1). These risk factors include hypertension, diabetes, obesity, and dyslipidemia.

Cardio-metabolic diseases constitute a major proportion of morbidity and mortality resulting from NCDs. The pathways for the development of the common cardio-metabolic diseases, especially CVDs that are set during development, are also interlinked (2). The development of cardio-metabolic diseases occurs slowly and progressively and thus demands a life-course approach of healthcare interventions (3). Despite these presumptions, the progressive development of the main cardio-metabolic conditions has not been well studied in sub-Saharan African settings.

The relationships between cardio-metabolic diseases and their risk factors have been studied well in many developed countries. For instance, the coexistence of diabetes and hypertension and its additive effects to increase the risk of cardiovascular events have been explored among large cohorts in different settings (4). Similarly, studies have examined the interlinkages between obesity and diabetes, hypertension and obesity, and hyperlipidemia and obesity (5–7). The associations between the anthropometric and clinical markers of cardio-metabolic conditions have also been evaluated in several settings in Western populations.

However, evidence about the interrelationships among NCDs in general and cardio-metabolic diseases in particular is very limited in sub-Saharan Africa. Most of the existing studies address the prevalence of common CVDs and their risk factors (8). More in-depth analysis of how the different factors interact to impact on the emergence of cardio-metabolic diseases is lacking. Studies that use more objective and valid measures of markers of cardio-metabolic diseases are also limited. In addition, most studies are small scale and tend to over-represent the real situation (9). Consequently, the evidence about cardio-metabolic diseases in urban poor settings of sub-Saharan Africa is inadequate for mitigation of the impacts of these conditions.

Therefore, this study has explored the patterns and interlinkages between the main cardio-metabolic markers using data from a large population-based cross-sectional study from urban poor settings in Nairobi, Kenya (10). In addition to filling the evidence gap related to analysis of interrelationships between cardio-metabolic conditions in sub-Saharan African settings, the findings will continue to inform the development and implementation of integrated approaches to the prevention and control of cardio-metabolic diseases in these and other similar settings (11).

## Methods

### Data source

Data were collected in two slums of Nairobi, Kenya – Viwandani and Korogocho – that are under surveillance within the Nairobi Urban Health and Demographic Surveillance System (NUHDSS) between May 2008 and April 2009. Details of the operation of the NUHDSS have been published elsewhere (12). We used data collected from 5,190 study participants as part of CVD risk assessment in the urban slums of Nairobi, Kenya. Twenty-one interviewers, who had been trained for 10 days, collected information using a structured questionnaire about basic demographic variables and risk factors of cardiovascular conditions. Weight, height, waist circumference, hip circumference, blood pressure, random blood glucose, total blood cholesterol, and triglyceride levels were also measured. Five commonly used clinical markers of cardio-metabolic conditions were considered in this analysis. These were waist circumference, blood pressure, random blood glucose, total blood cholesterol, and triglyceride levels.

### Measurement equipment

Waist circumference was measured using SECA (Hamburg, Germany) 201 circumference measurement tape. Three blood pressure measurements were conducted using an Omron (Kyoto, Japan) M6 blood pressure machine. The average of the second and the third blood pressure measurements were used in the analysis. ACCU-CHEK (Basel, Switzerland) Glucose, Cholesterol and Triglycerides (GCT) monitors and test strips were used for the measurement of random blood sugar, total blood cholesterol, and triglyceride levels.

### Definition of cardio-metabolic markers

Mean arterial pressure was computed as the sum of 2/3 diastolic blood pressure and 1/3 systolic blood pressure. Pulse pressure was calculated as the difference between systolic blood pressure and diastolic blood pressure. Increased values for systolic, diastolic, and mean arterial pressure were values above 140, 90, and 106 mmHg, respectively. With regard to random blood sugar levels, values greater than 140 mg/dl were considered hyperglycemic conditions and those greater than 200 mg/dl were taken as indicators of diabetes. Waist circumference values greater than 102 cm (men) and 88 cm (women) were taken as markers of central obesity. Hypercholesterolemia and hypertriglyceridemia were defined by total blood cholesterol of greater than 200 mg/dl and triglyceride levels of greater than 180 mg/dl, respectively (13–15).

### Data analysis

Prevalence and patterns of the five cardio-metabolic markers were described using means, standard deviations (SDs), and proportions. Multiple occurrence of the five cardio-metabolic markers was explored using summated scores of the markers. Sampling probability weight was computed using the size of the stratum in the NUHDSS database as denominator, and response probability was calculated using the total number sampled per stratum as denominator. A composite weight taking both sampling and response weights into account was applied to prevalence estimates when needed. Logistic regression models were used to assess the binary association between the five cardio-metabolic markers. The associations between the markers are determined using odds ratios (ORs). The association between the presence of multiple markers and participants’ characteristics (age, sex, education, and working status) was also explored using ORs. CIs of ORs were also used to determine the statistical significance of the OR. Numeric and tabular summaries are used to present the findings.

### Ethics statement

The study protocol was approved by the Kenya Medical Research Institute/National Ethical Review Committee (NON-SSC Protocol No. 339). Participants provided written consent to participate in the study. Those participants who had agreed to be interviewed signed a consent form to show that they agreed to participate in the study. The ethics committee approved the consent procedure along with the protocol and data collection tools.

## Results

### Background characteristics

A total of 5,190 individuals were included in this analysis. Of these 2,794 (53.8%) were males and the remaining 2,396 (46.2%) were females. The majority of respondents (2,899; 55.8%) were younger than age 45, while 673 (12.9%) were older than 60.

### Blood pressure levels

The mean (SD) systolic and diastolic blood pressures in the study population were 124.4 (19.7) mmHg and 76.5 (12.1) mmHg, respectively. The mean (SD) arterial pressure (MAP) was 92.5 (13.7) mmHg, whereas the mean pulse pressure was 47.9 (13.4) mmHg. There were no remarkable differences in mean blood pressure levels between men and women. However, the mean (SD) MAP levels had an obviously increasing trend across age groups: 89.0 (11.0) mmHg in those younger than 45, 96.0 (15.2) mmHg in those aged 45–60, and 99.0 (15.5) mmHg in those older than 60.

Of all the study population included in this analysis, 728 (14.3%) had systolic blood pressure above 140 mmHg, whereas 524 (10.3%) had diastolic blood pressure above 90 mmHg. Both systolic blood pressure above 140 and diastolic blood pressure above 90 were found in 386 (7.5%) of the respondents. Increased systolic blood pressure (>140 mmHg) alone was detected in 342 (6.7%) individuals, whereas increased diastolic blood pressure (>90 mmHg) alone was present in 138 (2.7%) participants. Overall, 866 (17.0%) of the study participants had increased blood pressure in at least one of the readings.

When MAP was examined, 632 (12.4%) had above-normal MAPs (13.7% in women and 11.3% in men). Analysis of pulse pressure among the study subjects showed that 1,467 (28.8%) had a pulse pressure of 40 mmHg or less and 645 (12.7%) had a pulse pressure of greater than 60 mmHg. Higher pulse pressure was more prevalent in men (13.6%), whereas low pulse pressure was more prevalent among women (36.1%). The prevalence of high pulse pressure among those older than 60 was 39.4%, whereas the prevalence of low pulse pressure among those younger than 45 was 34.6%. Details are shown in Table 1.

**Table 1:** Prevalence of increased blood pressure measurements

	Women	Men	Both sexes
Systolic blood pressure	356 (15.2%)	372 (13.6%)	728 (14.3%)
Diastolic blood pressure	283 (12.1%)	241 (8.8%)	524 (10.3%)
Mean arterial pressure	322 (13.7%)	310 (11.3%)	632.5 (12.4%)
Pulse pressure	274 (11.7%)	371 (13.6%)	645 (12.7%)

### Random blood glucose levels

The mean (SD) random blood glucose level in the study population was 87.1 (30.6) mg/dl. This was 88.9 (32.4) in women and 85.5 (28.8) in men. The mean (SD) blood sugar levels increased with age. It was 84.7 (23.2) in those younger than 45, 89.7 (36.3) in those aged 45–60, and 91.1 (41.3) in those older than 60.

Among the study participants, 183 (3.65%) had hyperglycemia, a random blood sugar of more than 140 mg/dl. Women had a higher prevalence of hyperglycemia as compared to men (3.75 vs. 3.33%). The prevalence of diabetes was also higher among women than men (1.26 vs. 0.56%). The prevalence of diabetes among those younger than 45, aged 45–60, and older than 60 were 0.21, 1.67 and 1.88%, respectively. As shown in Table 2 the prevalence of hyperglycemia in both sexes increases with age.

**Table 2:** Prevalence of hyperglycemia by age and sex

Age group (years)	Women	Men	Both sexes
Younger than 45	28 (1.17%)	28 (1.00%)	56 (2.00%)
45–60	36 (1.50%)	48 (1.72%)	84 (5.38%)
Older than 60	26 (1.08%)	17 (0.61%)	43 (6.73%)
All age groups	90 (3.75%)	93 (3.33%)	183 (3.65%)

### Total blood cholesterol levels

The mean (SD) level of total blood cholesterol was 4.35 (0.83) mmol/l. Men had a comparatively lower mean total blood cholesterol level as compared to women (4.31 vs. 4.39). Analysis of mean cholesterol levels by age indicated that mean total blood cholesterol levels increased with age: 4.25 in those younger than 45, 4.45 in those aged 45–60, and 4.53 in those older than 60.

Considering a total blood cholesterol level of <200 mg/dl as normal, 614 (13.4%) of the study population had increased total blood cholesterol levels. The prevalence of raised total blood cholesterol level was higher in women (15.2%) than in men (11.8%). There exists a strong increasing trend of prevalence of raised total blood cholesterol levels by age group. Those study participants younger than 45 had a prevalence of 9.8%, whereas those aged 45–60 had a prevalence of 15.9%. More than one-fifth (21.8%) of those older than 60 had increased total blood cholesterol levels.

### Triglyceride levels

The mean (SD) triglyceride level in the study population was 1.71 (1.01) mmol/l. Women and men had mean (SD) triglyceride levels of 1.73 (1.11) and 1.70 (0.93), respectively. Unlike the patterns observed in the other cardio-metabolic markers, the age patterns of triglyceride levels were a bit different. Those between the age of 45 and 60 had a higher mean triglyceride level (1.80 mmol/l) as compared to those older than 60 (1.73 mmol/l) and those younger than 45 (1.65 mmol/l).

Triglyceride levels of <180 mg/dl are considered normal; 830 (18.8%) of the study population had raised triglyceride levels. The prevalence of hypertriglyceridemia was 19.3% in women and 18.3% in men. In line with the trends in the mean triglyceride levels, the prevalence of hypertriglyceridemia was high in those aged 45–60 (22.7%) as compared to those older than 60 (18.1%) and younger than 45 (16.7%).

### Central obesity

In the study population, the weighted prevalence of overweight (body mass index between 25 and 30 kg/m^2^) and general obesity (body mass index >30 kg/m^2^) were 20.5 and 7.8%, respectively. The mean (SD) waist circumference of the study population was 83.2 (10.6) to 85.6 (11.7) cm in women and 81.2 (9.1) cm in men. In women, the mean (SD) waist circumference values were 83.4 (11.0) in those younger than 45, 89.0 (11.6) in those aged 45–60, and 88.7 (12.7) in those older than 60. Using the American Heart Association's cutoff points of waist circumference for defining obesity, 948 (18.8%) of the study participants had obesity. This was 38.2% in women and 2.4% in men. The prevalence trends in women by age shows that prevalence of central obesity increases with age in women. Those women older than 60 had a prevalence of 25%.

### Prevalence of raised levels of multiple cardio-metabolic markers

The weighted prevalence of individual cardio-metabolic disease markers are shown in Table 3. The prevalence of increased level of at least one of the five cardio-metabolic makers was found to be 47.3%. This figure was higher in women (59.7%) than in men (37.1%). About 30% of the study population had raised levels in any one of the five cardio-metabolic markers. Similar to the pattern of most of the cardio-metabolic markers, the prevalence of raised levels of at least one of the five cardio-metabolic markers increased with age. It was 38.7, 54.8, and 62.6% among those younger than 45, aged 45–60, and older than 60, respectively.

**Table 3:** Weighted prevalence of increased cardio-metabolic markers by sex

	Men (%)	Women (%)	Total (%)
Hypertension	7.2	6.6	7.0
Hyperglycemia	2.5	2.4	2.5
Hypercholesterolemia	9.2	11.8	10.3
Hypertriglyceridemia	18.2	16.0	17.3
Central obesity	1.4	29.3	12.3

The association between the presence of raised levels of multiple (two or more) cardio-metabolic markers illustrates that men were less likely to have raised levels of multiple markers as compared to women (OR = 0.24; 95% confidence interval [CI]: 0.19, 0.29). Participants aged 45–60 (OR = 3.17; 95% CI: 2.59, 3.89) and older than 60 (OR = 4.15; 95% CI: 3.18, 5.42) were at a higher risk of having raised levels of multiple markers as compared to those younger than 45. On the other hand, participants with no formal education were less likely to have raised levels of multiple risk factors (OR = 0.74; 95% CI: 0.58, 0.96). There was no statistically significant association between the presence of raised levels of multiple risk factors and the working status of the participant.

The weighted prevalence of these is shown in Table 4.

**Table 4:** Weighted prevalence of raised levels of multiple cardio-metabolic markers

	Men (%)	Women (%)	Total (%)
No raised marker	67.5	50.9	61.2
At least one raised marker	49.1	32.5	38.8
Any one raised marker	26.6	32.8	28.9
Two raised markers	4.9	11.6	7.5
Three or more raised markers	0.99	4.62	2.4

### Interlinkage between the cardio-metabolic markers

Assessment of the associations among the five cardio-metabolic markers considered for this study revealed the existence of a statistically significant positive association in all 10 combinations of the five cardio-metabolic markers except between blood glucose and blood cholesterol, and blood pressure and blood cholesterol. The strongest associations were the association between blood glucose and waist circumference and blood glucose and blood pressure. The associations between blood pressure and waist circumference and blood glucose and triglycerides were also strong. The OR and 95% CIs for the interlinkage between the five cardio-metabolic markers are shown in Table 5.

**Table 5:** Association between the five cardio-metabolic markers (adjusted for age and sex)

SN	Cardio-metabolic markers	Odds ratio	95% confidence interval
2	Blood glucose – central obesity	4.07	(2.70, 6.15)
1	Blood pressure – blood glucose	2.90	(2.07, 4.06)
3	Blood pressure – central obesity	2.27	(1.80, 2.86)
4	Blood glucose – triglycerides	2.22	(1.59, 3.10)
5	Triglycerides – central obesity	2.22	(1.79, 2.74)
6	Blood pressure – triglycerides	1.72	(1.39, 2.13)
87	Blood cholesterol – central obesity	1.38	(1.09, 1.74)
98	Blood pressure – cholesterol	1.23	(0.97, 1.56)^ns^
9	Blood cholesterol – blood glucose	0.97	(0.63, 1.48)^ns^

SN, serial number; ns, not significant.

The findings reveal that blood glucose has a strong association with all other cardio-metabolic markers except total blood cholesterol. Further analysis of the association between cardio-metabolic markers with body shape (using waist-to-hip ratio, WHR) showed that all four markers had a statistically significant association with WHR. These were 1.61 (95% CI: 1.15, 2.25) for blood glucose; 1.55 (95% CI: 1.28, 1.87) for blood pressure; 1.38 (95% CI: 1.17, 1.62) for triglycerides; and 1.83 (95% CI: 1.52, 2.22) for total cholesterol.

## Discussion

This study has revealed that nearly 40% of adults 18 years and above in urban slum settings have at least one increased cardio-metabolic marker. Almost 10% of the same population group has two or more increased cardio-metabolic markers. The results of this study have also demonstrated a strong level of interlinkage among the five cardio-metabolic markers. This finding shows that an individual with an increased level of one of the cardio-metabolic markers is more likely to have an increased level of the other cardio-metabolic markers, as well.

The association between hyperglycemia and hypertension is also similar in other studies. A study conducted in India showed a significant correlation between fasting blood sugar and diastolic blood pressure (16). Hence, the co-prevalence of hypertension and hyperglycemia especially in young adults with these conditions requires joint prevention and control interventions before full-blown disease and complications occur. Similarly, in this study, the association between hypertension and hyperglycemia was the strongest form of association among the biomarkers included in the analysis.

The association between blood glucose and central obesity in this study was the second strongest form of association. Participants with hyperglycemia are at a risk more than three times higher of having abdominal obesity as compared to those without hyperglycemia. In a study conducted in Japanese study participants, the association between hyperglycemia and obesity was statistically significant across all age groups and in both men and women. This association was also found to decline with age (17). In this regard, the current study in an urban poor setting found similar levels of associations between hyperglycemia and obesity as compared to a study in a developed country.

The findings of this study have also shown that hypertension and central obesity are strongly linked. Hypertensive individuals are more likely to be overweight and vice versa. This has been the case in many other studies since the 1960s (18). Obesity itself increases the risk of hypertension and this has been the subject of recent reviews (19). In this regard, the current study corroborates evidence of the linkage between hypertension and obesity from an urban poor setting in sub-Saharan Africa.

The association between blood glucose and blood cholesterol is also significantly high in the presented findings. The mechanisms linking hyperglycemia and hypercholesterolemia have been well described (20, 21). However, with such a strong association between hyperglycemia and hypercholesterolemia, the findings of this study warrant a concerted effort to tackle hyperglycemia and hypercholesterolemia among those adults with a higher risk in urban poor settings.

This study has described the interlinkage between the five main cardio-metabolic markers in an urban poor setting in Africa. However, the main limitation with this study was its cross-sectional design. All the markers were measured once and at the same time. Hence, establishing the direction of the association between these markers was difficult. Data on Low Density Lipoprotein cholesterol (LDL-C) was not collected in the main survey. Moreover, the study measured random blood sugar, which may have had higher variability as compared to fasting blood sugar and HbA1C.

## Conclusions and recommendations

This study assessed the interlinkage between five common markers of cardio-metabolic conditions. It was found that women have a highly disproportionate prevalence of hypercholesterolemia and central obesity. The analysis also found that nearly 40% of the study population had increased levels of at least one of the five cardio-metabolic markers, with about 10% having increased levels in multiple cardio-metabolic markers.

The associations between four pairs of cardio-metabolic markers were found to be significantly high. These were the associations between blood glucose and blood pressure, blood glucose and central obesity, blood pressure and central obesity, and blood glucose and triglycerides. All other associations, except the association between blood glucose and blood cholesterol, were also statistically significant. The findings suggest higher levels of co-occurrence of cardio-metabolic conditions in the study population and the need for integrated approaches in the future.
